# Comparative metagenomics study reveals pollution induced changes of microbial genes in mangrove sediments

**DOI:** 10.1038/s41598-019-42260-4

**Published:** 2019-04-05

**Authors:** Yingdong Li, Liping Zheng, Yue Zhang, Hongbin Liu, Hongmei Jing

**Affiliations:** 1 Division of Life Science, The Hong Kong University of Science and Technology, Kowloon, China; 2 CAS Key Laboratory for Experimental Study under Deep-sea Extreme Conditions, Institute of Deep-sea Science and Engineering, Chinese Academy of Sciences, Sanya, China

## Abstract

Mangrove forests are widespread along the subtropical and tropical coasts. They provide a habitat for a wide variety of plants, animals and microorganisms, and act as a buffer zone between the ocean and land. Along with other coastal environments, mangrove ecosystems are under increasing pressure from human activities, such as excessive input of nutrients and toxic pollutants. Despite efforts to understand the diversity of microbes in mangrove sediments, their metabolic capability in pristine and contaminated mangrove sediments remains largely unknown. By using metagenomic approach, we investigated the metabolic capacity of microorganisms in contaminated (CMS) and pristine (PMS) mangrove sediments at subtropical and tropical coastal sites. When comparing the CMS with PMS, we found that the former had a reduced diazotroph abundance and nitrogen fixing capability, but an enhanced metabolism that is related to the generation of microbial greenhouse gases via increased methanogenesis and sulfate reduction. In addition, a high concentration of heavy metals (mainly Zn, Cd, and Pb) and abundance of metal/antibiotic resistance encoding genes were found in CMS. Together, these data provide evidence that contamination in mangrove sediment can markedly change microbial community and metabolism; however, no significant differences in gene distribution were found between the subtropical and tropical mangrove sediments. In summary, contamination in mangrove sediments might weaken the microbial metabolisms that enable the mangrove ecosystems to act as a buffer zone for terrestrial nutrients deposition, and induce bioremediation processes accompanied with an increase in greenhouse gas emission.

## Introduction

Mangrove ecosystems are widely distributed in the transition between land and sea, particularly along the estuaries and coastlines in subtropical and tropical regions. The microorganisms that inhabit mangrove sediments play a critical role in the biogeochemical cycling of carbon, nitrogen, phosphorus, as well as the deposition of heavy metals from adjacent land and fluvial imports, through metabolic processes^1,2^. In recent years, the increasing level of contamination in mangrove sediments has started to pose a serious threat to mangrove forests, with plants disappearing at a rate estimated to be ~1–2% globally per year^3^. Although mangroves act as a natural sewage treatment plant, exhibiting a robust ecosystem restoration capacity^4,5^, the ever increasing levels of contaminants are now overloading mangrove sediments, and this influences their restoration and nutrient cycling capacity by affecting the sediment-inhabiting microorganisms^6–9^.

The effects of anthropogenic nutrients and pollutants on the microflora in mangrove sediments have been widely studied^10–12^. It has been demonstrated that contamination of the mangrove sediment can significantly promote the diversity of the microbial community as a whole, but at the same time it has a negative impact on some specific groups of microorganisms^13,14^. For example, acetoclastic/methylotrophic methanogens exhibited a decreased level of diversity in a contaminated mangrove in Singapore^6^. In addition, metals are retained in mangrove sediments rather than moving to adjacent environments, but this also markedly influences the autochthonic microbial community^15^. For example, both metals and metalloids have been shown to contribute to the abundance and distribution of ammonia-oxidizing archaea and beta-proteobacteria, both of which are crucial to the nitrogen cycle processes in mangrove sediments^1,16,17^. The fact that polycyclic aromatic hydrocarbon- (PAH-) and phenanthrene polluted mangrove sediments can also increase growth of specific microbial degraders, which illustrates the strong adaptation and bioremediation ability of mangrove sediment-residing microorganisms^18–21^. “To further confirm the distribution of microbial genes in mangrove sediment, researchers had applied metagenomic approach in many different regions recently. For instance, Andreote *et al*. had investigated the microbial participants in sulfur and nitrogen cycle in Brazilian mangrove sediment^22^; Imchen *et al*. studied the variation of microbial taxon and metabolic genes across diverse mangrove sediment system^23^; the prevalent distribution of heavy metals and antibiotic resistome in mangrove sediments at India, Brazil and Saudi Arabia was also reported^24^. However, the lack of a fundamental level of understanding of the microorganism-related metabolic capability in pristine and contaminated mangrove sediments hinders our determination of the carrying capacity of the ecosystem, as well as the capability of the microbiome in responding to a further increase in pollution in mangrove sediments.

Mangroves located in the northern and southern parts of Hainan Island (China) are representative of subtropical and tropical habitats, respectively^25,26^. Located in the northern part of Hainan Island, Dongzhai harbor in Haikou city has one of the largest natural mangrove forests in China and it is the first to have been made into a nature reserve; however, the local aquaculture industry has resulted in severe eutrophication in the offshore part of reserve, while the inshore part is relatively pristine. In contrast, the mangroves in Sanya city, located at the southern tip of Hainan Island, have been partially contaminated by both pre-treated and untreated domestic sewage as well as by industrial wastewater in inshore part, while the offshore part is relatively pristine. Thus, the mangroves in Dongzhai and Sanya are ideal sites for us to investigate the metabolic capacity of microorganisms in both contaminated and uncontaminated mangrove sediments under different climatic conditions. By applying metagenomic approach, researchers had already investigated

In this study, we explored the community structure and metabolism capacity of the microorganisms in the heavily polluted and relatively pristine mangrove sediments in both tropical Sanya and subtropical Haikou using a metagenomic approach.

## Results

### Environmental conditions and methane flux

As shown in supplementary Table S1, the concentration of total phosphorus (TP) and total organic carbon (TOC) were higher in subtropical mangrove sediment than in the tropical sediments. On the other hand, the tropical mangrove sediment had a higher pH and a higher concentration of ammonia than the subtropical mangrove sediment. In both the subtropical and tropical mangroves, the contaminated sediments exhibited higher concentrations of nitrogen source (Supplementary Table S1).

With regards to the amounts of metals in the sediment, the concentration of Zn was higher in the CMS than in the PMS in both subtropical (i.e., with 3577.11 mg/kg in contaminated mangrove sediment of Haikou (HKC) and 2687.74 mg/kg in pristine mangrove sediment of Haikou (HKP)) and tropical (i.e., with 371.14 mg/kg in contaminated mangrove sediment of Sanya (SYC) and 205.53 mg/kg in pristine mangrove sediment of Sanya (SYP)) samples. In addition, the concentration of Zn in the subtropical sites was an order magnitude higher than that in the tropical sites. Similarly, the amounts of Cd (0.37 mg/kg in HKC, 0.27 mg/kg in HKP, 0.16 mg/kg in SYC, and 0.11 mg/kg in SYP) and Pb (18.57 mg/kg in HKC, 16.11 mg/kg in HKP, 22.94 mg/kg in SYC, and 7.26 mg/kg in SYP) were also all higher in the CMS than in the PMS (Table S1). In addition, the methane flux across the mangrove sediment-atmosphere interface was higher in the CSM (0.286~0.578 mg·m^−2^ h^−1^) than in the PMS (−0.143~−0.017 mg·m^−2^ h^−1^), and a higher methane flux was measured in the subtropical mangrove sediment than in the tropical sites (Table S1).

### Microbial community composition

After assembly of the quality controlled short reads, a total of 810,027 contigs (>400 bp) were obtained, ranging from 172,238 to 231,903 contigs in the four samples (Supplementary Table S2). The detail information of sequence reads before and after QC, and annotation was summarized into Table S3. As the average coverage of assembled contigs in each sample was >18, this suggests that the sequencing well covered the genomic information in samples. The coverage number of all identified functional genes and SSU rRNAs were normalized to the sequencing size of each sample to achieve comparison across samples.

**Figure 1:**
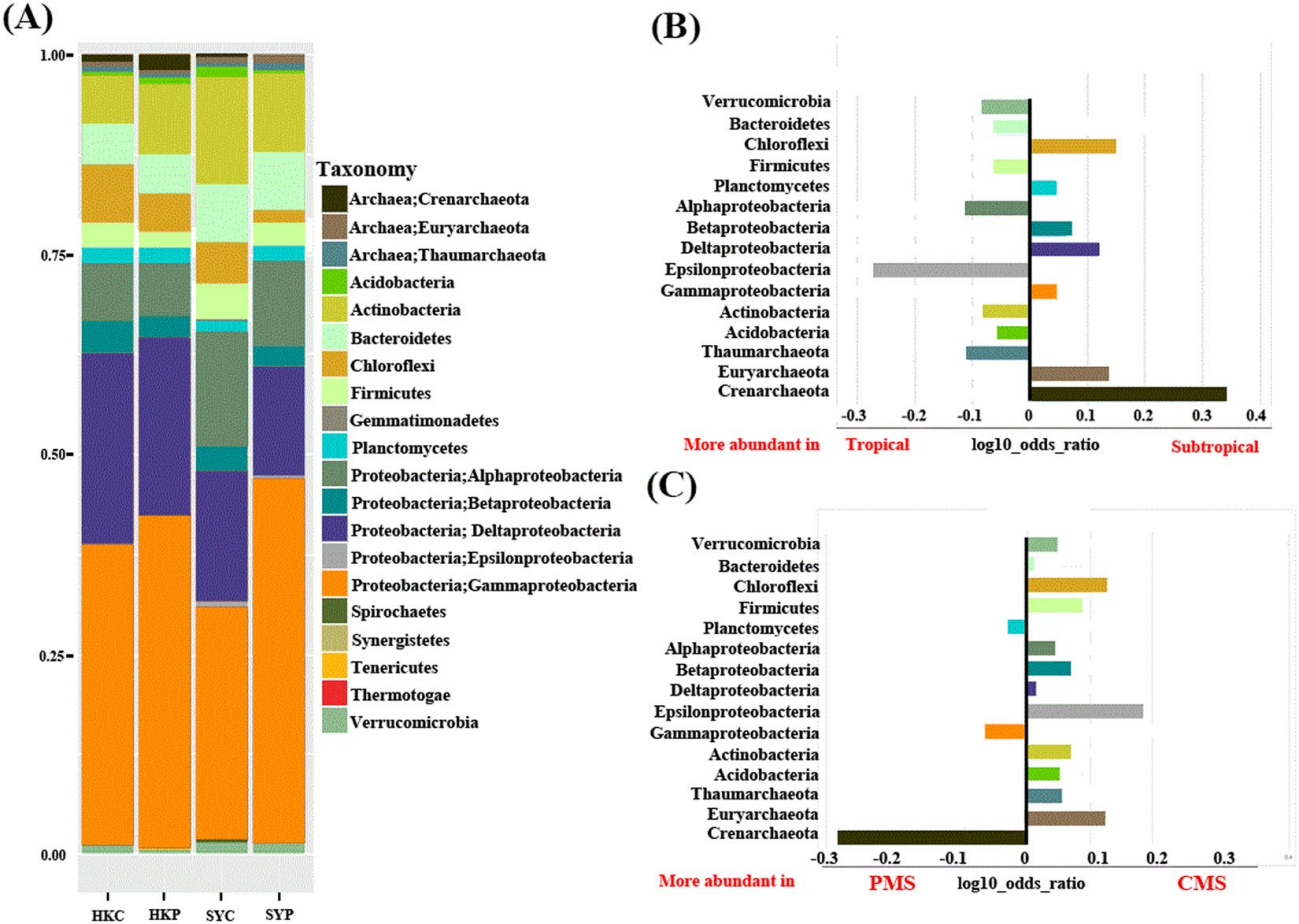
(**A**) The composition of the microbial communities in the various sampling sites at the phylum level, (**B**) and a differential analysis between the tropical and subtropical regions; (**C**) and between the pristine (PMS) and contaminated (CMS) regions. For the differential analysis, values are the base-10 logarithm of the odds ratio. The positive and negative values indicate taxa that are more in (**B**) and (**C**), respectively.

Information of microbial community was identified by screening SSU rRNAs from the assembled contigs in each sample. Twenty prokaryotic phyla and major subclasses within Proteobacteria and Archaea were found in the samples (Fig. 1). In general, three subclasses of *Proteobacteria* (i.e., *Deltaproteobacteria, Alphaproteobacteria* and *Gammaproteobacteria)*, were widely distributed among the samples. The most abundant, i.e., *Gammaproteobacteria*, accounted for more than 25% of all the phyla across the four samples, but they showed a marked decrease in the CMS (Fig. 1A,C). The highest and lowest relative abundance of *Deltaproteobacteria* was found in the HKC (14.4%) and SYP (12.3%). In addition, the relative abundance of *Alphaproteobacteria* ranged from 4.5% to 8.3% across all the samples, with the highest and lowest values found in the SYC and HKP, respectively, and this class was markedly enriched in the tropical mangrove sediment with a log10 odds ratio of −0.1 (Fig. 1B). Microbial taxa that were significantly enriched (i.e., with a log10 odds ratio >0.1 or <−0.1) were also found in the samples. For example, *Chloroflexi*, *Euryarchaeota*, *Deltaproteobacteria* and *Crenarchaeota* were highly enriched in the subtropical samples; whereas *Epsilonproteobacteria*, *Alphaproteobacteria*, and *Thaumarchaeota* were more abundant in the tropical samples (Fig. 1B). Furthermore, *Epsilonproteobacteria*, *Chloroflexi*, and *Euryarchaeota* were enriched in the CMS; whereas *Crenarchaeota* were more abundant in the PMS (Fig. 1C).

**Figure 2:**
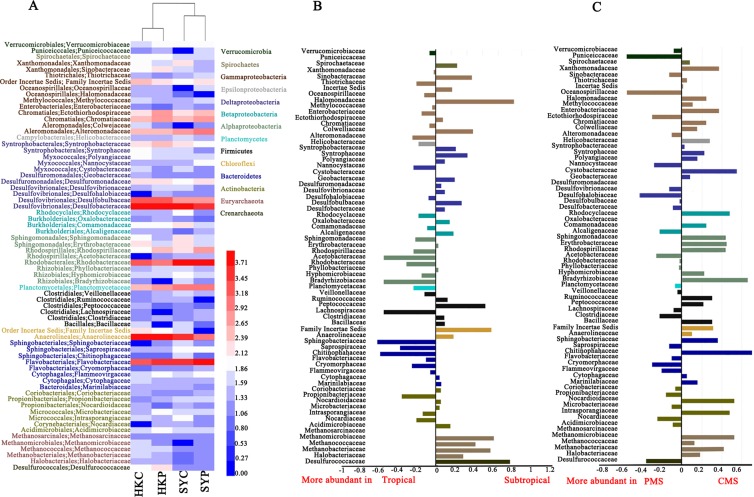
(**A**) The composition of the microbial community at the family level, (**B**) and a differential analysis between the tropical and subtropical regions; (**C**) and between the pristine (PMS) and contaminated (CMS) regions. For the differential analysis, values are the base-10 logarithm of the odds ratio. The positive and negative values indicate taxa that are more in (**B**) and (**C**), respectively.

At the family level, the microbial taxa also showed distinct distribution patterns in the different regions (i.e., subtropical vs tropical (Fig. 2B)) and at the different levels of contamination (CMS vs PMS (Fig. 2C)). For example, *Acetobacteraceae*, *Bradyrhizobiaceae*, *Sphingobacteriaceae*, *Lanchnospiraceae*, and *Chitinophahaceae* were significantly enriched (i.e., with an odds ratio <−0.6) in the tropical mangrove sediment, whereas *Peptococcaceae*, *Halomonadaceae*, *Desulfurococcaceae, Methanobacteriaceae, Methanococcaceae, Methanomicrobiaceae* were all more abundant (with an odds ratio >0.6) in the subtropical mangrove sediment (Fig. 2B). A comparison of the microbial taxa found in the contaminated and pristine mangrove sediments revealed that *Bradyrhizobiaceae*, *Erythrobacteraceae*, *Sphingomonadaceae*, *Rhodocyclaceae*, *Cystobacteraceae*, *Enterobacteriaceae*, *Xanthomonadaceae*, *Methanosarcinaceae*, and *Methanobacteriaceae* were all enriched (with an odds ratio >0.4) in the CMS, while *Desulfohalobiaceae*, *Oceanospirillaceae*, and *Puniceicoccaceae* were more abundant (with odds ratios of −0.42, −0.58 and −0.57, respectively) in the PMS (Fig. 2C).

### Nutrient metabolism and metal resistance encoding genes

**Figure 3:**
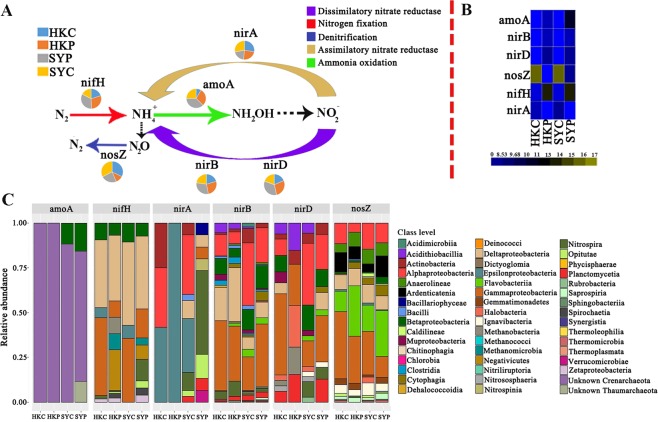
(**A**) Pie charts and schematic to show the contribution of the various samples (HKC, HKP, SYP and SYC) to different aspects of nitrogen metabolism. The different colored arrows represent different parts of the metabolic pathway (as shown in the key in the upper right), whereas the black dashed arrows represent the pathways and genes that were omitted. The pie charts represent the relative abundance of key nitrogen metabolism-related genes, which was calculated by dividing the sum of a gene’s coverage in one sample by the sum of the gene’s coverage in all the samples. (**B**) The absolute abundance of each gene was double square rooted. (**C**) The microbial taxa of the genes and its relative abundance in each sample.

**Figure 4:**
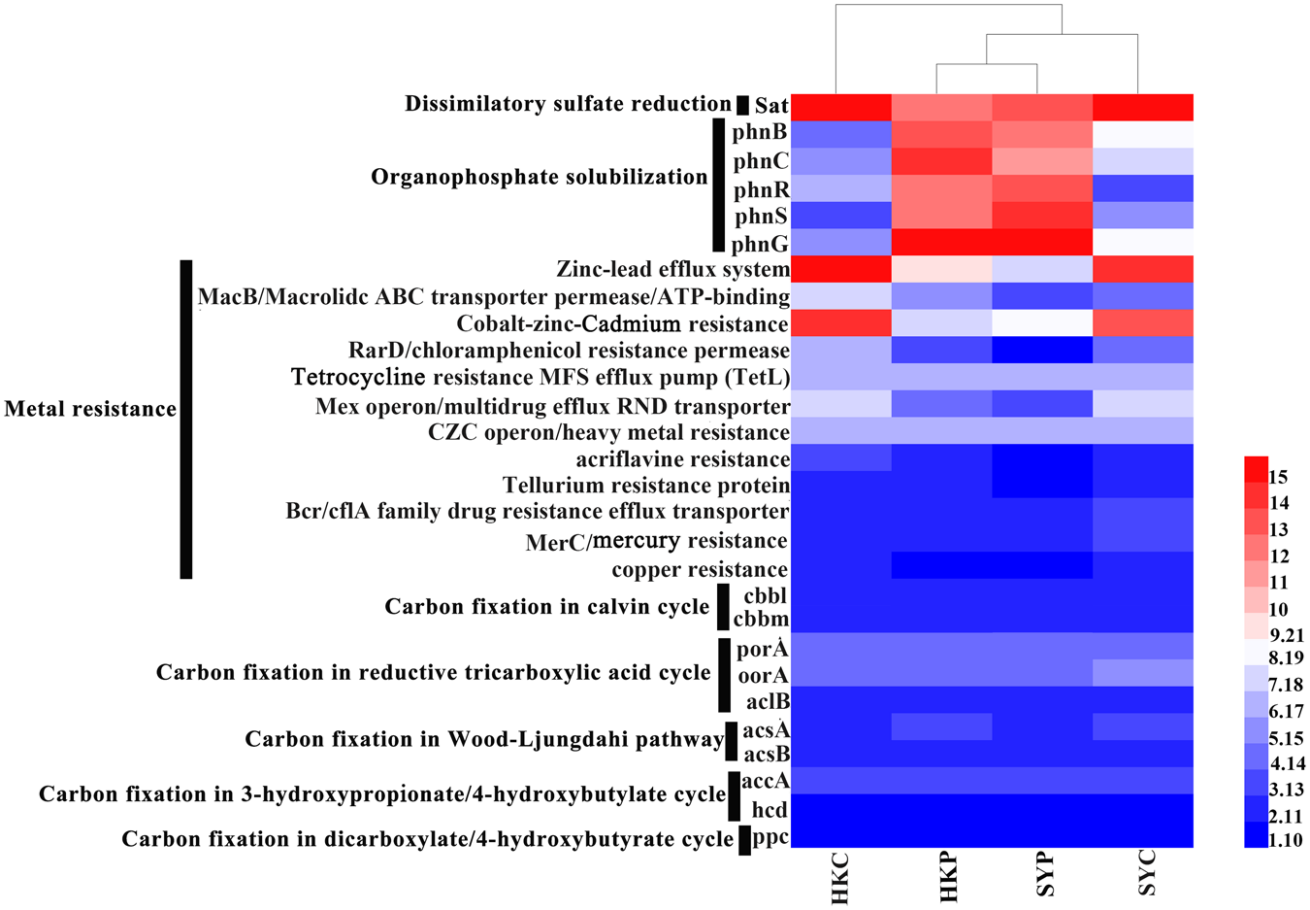
The absolute abundance of organophosphate solubilization and metal resistance genes. Each value was double squared rooted.

The nutrient metabolism-related genes were distributed differently among the various samples. A higher abundance of the nitrogen fixation gene (*nifH)* was found in the subtropical region (HKC and HKP), whereas key genes in ammonia oxidation (*amoA*), assimilatory nitrate reduction (*nirA*), dissimilatory nitrate reduction (*nirD*), and denitrification (*nosZ*) were more abundant in the tropical region (SYC and SYP) (Fig. 3A). In addition, *nifH*, *amoA*, and the dissimilatory nitrate reduction genes (*nirB* and *nirD*) exhibited a higher abundance in the pristine samples; whereas *nirA* and *nosZ* were more abundant in the contaminated samples (Fig. 3A,B). The organophosphate solubilizing genes were more abundant in the PMS (HKP and SYP), whereas genes encoding the zinc-lead efflux system, cobalt-zinc-cadmium resistance, and dissimilatory sulfate reduction were more abundant in the CMS (HKC and SYC) (Fig. 4). In addition, although our results indicated that there was no difference in the distribution of carbon fixation genes in across the samples, the preferred carbon fixation pathway (i.e., Carbon fixation in reductive tricarboxylic acid cycle) was still being found (Fig. 4).

To distinguish between the affiliated microbial taxa of each nitrogen-related metabolism gene, the microbial taxa were extracted using the MEGAN software, and the percentage they accounted in each gene was plotted (Fig. 3C). A higher abundance of *Gammaproteobacteria* (affiliated with *nirD* and *nosZ)*, and *Alphaproteobacteria* (affiliated with *nirB* and *nirD)* were found in the subtropical and tropical sediments, respectively. In addition, we also found that within the *nosZ*-affiliated microbial taxa, *Pseudomonadales*, which is a typical heterotrophic denitrifying bacteria, was abundant in the CMS (Fig. S1). However, diazotrophs were more diverse in the PMS than in the CMS; for example, *Spirochaetia*, *Methanomicrobia*, and *Dehalococcoidia* all uniquely inhabited the PMS (Fig. 3C). The most prevalent diazotroph, *Deltaproteobacteria*, contributed >25% of all of the *nifH* genes in each sample; this was followed by *Gammaproteobacteria*, which exhibited a much higher relative abundance of *nifH* in the contaminated samples than in the uncontaminated samples, with relative abundances ranging from 24.2% to 25.3% in the CMS. The *nirA* affiliated microbial taxa showed a higher level of diversity in the CMS than the PMS. However, the *amoA* affiliated taxa exhibited a lower diversity of microbial affiliation across all the samples. For example, unknown *Crenarchaeota* were the only contributor of *amoA* gene in the subtropical samples, and it was the major contributor in the tropical samples. In addition, *Betaproteobacteria* contributed 15.4% and 18.2% in the SYC and SYP, respectively, whereas a small proportion of *amoA* gene in the SYP was affiliated with *Thaumarchaeota*.

### Methane metabolism encoding genes

**Figure 5:**
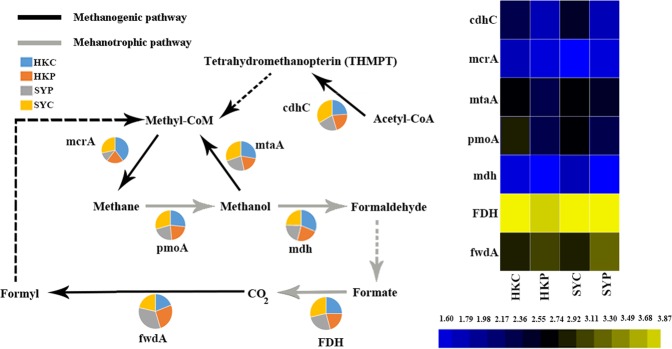
(**A**) Pie charts and schematic to show the contribution of the various samples (HKC, HKP, SYP and SYC) to different aspects of methane metabolism. The pie charts show the relative abundance of key methane metabolism related genes, which was calculated by dividing the sum of one gene’s coverage in an individual sample by the sum of the same gene’s coverage in all the samples. The dashed lines indicate where other genes involved in the pathway were omitted. (**B**) The absolute abundance of each gene was double square rooted.

The abundance of essential microbial methane generation and utilization genes was investigated (Fig. 5). A comparison of the methanogenic bacteria in the CMS and PMS showed that key genes in heterotrophic methanogens, such as those generating methane with acetate (acetyl-CoA decarbonylase/synthase; *cdhC*) or methanol (methyltransferase; *mtaA*), were more abundant in the CMS (HKC and SYC). In contrast, formylmethanofuran dehydrogenase (*fwdA*), which is a key gene in autotrophic methanogens, was more abundant in the PMS (HKP and SYP). Essential genes within the metabolic pathway of methanotrophic bacteria (for example *pmoA* and *mdh*), were more abundant in the CMS (HKC and SYC). It was also noticeable that within the methanotrophic pathway, the gene functioning to generate carbon dioxide was more abundant in the CMS.

## Discussion

### Effect of contamination on the microbial community

Our results showed that contamination of mangrove sediment did shape the microbial community. It is known that diazotrophs are frequently influenced by the level of nutrients, the type of organic and inorganic compounds, and the presence of metals^27–29^. This might therefore explain why less abundant diazotrophs, such as *Rhodospirillacene* within the *Alphaproteobacteria* class^30^, and *Clostridiaceae* within the *Firmicutes* phylum^31^ (Fig. 2), were both found in the CMS when comparing with that in PMS. Although the usual deficit of oxygen together with an abundant amount of organic matter in mangrove sediments naturally selects for a number of anaerobic organisms, including methanogens, sulphate-reducing bacteria (SRB) and denitrifiers^32^, the marked enrichment of methanogens (*Methanomicrobiaceae*, *Methanosarcinaceae*, *Methanococcaceae*, and *Methanobacteriaceae*) and SRB bacteria (*Desulfovibrionaceae* and *Desulfobacteraceae*) in the CMS, reflects the impact of contamination on the microbial communities (Fig. 2). As methanogenic or sulfate-reducing conditions stimulate the degradation of organic contamination^33–35^, the enriched levels of methanogens and SRB in the CMS might be an effect of bioremediation. As mentioned above, the different contaminants in the CMS not only affects the distribution of diazotrophs, but also stimulates bioremediation-related microbial taxa. In previous study, researchers had found significant differences in microbial community and functions between ocean and terrestrial mangrove sediment^23^. Our study further identified the difference of microbial taxa in the sediments of the subtropical and tropical mangrove sediments, and the similar gene enrichment response to anthropogenic inputs of nutrients and pollutants.

### Nitrogen cycling within mangrove sediments

One of the main pollutants was inorganic nitrogen (Supplementary Table S1); thus, in contaminated mangrove sediments, the nitrogen source is more available for microbe to be assimilated into biomass when comparing with that in pristine mangrove sediment. The high abundance of denitrification and nitrogen assimilation encoding genes, in the CMS, together with the low abundance of nitrogen-fixing genes in the CMS (Fig. 3) suggests that nitrogen is not limited in the contaminated ecosystem, and thus nitrogen acquisition through nitrogen fixation is not critical. Similarly, an enrichment of denitrification encoding genes was also found in Brazilian contaminated mangrove sediments, which further supports the contamination could induce the microbial denitrification process^22^. The more abundant nitrogen fixation encoding genes in the pristine mangroves indicates that the microbes are struggling to achieve the same result, to assimilate nitrogen source into biomass, in the pristine sediments but more rely on atmospheric N_2_. This also indicates a faster resource recycle metabolism, since the energy for N_2_ fixation is mainly derived from the decomposition of dead leaves and roots in mangrove sediments^36,37^. The massive input of nitrate/nitrite and the high levels of heavy metals in the CMS might contribute to the observed decrease in the ammonia oxidation encoding genes, as these types of contamination are known to have a negative effect on the activity and abundance of ammonia oxidizers^38,39^.

In addition, the low level of oxygen in the CMS, which is shown by the increase both in methane flux and anaerobic methanogens (Fig. 2)^40,41^, might also lead to a decrease in the abundance of aerobic ammonia oxidizing microorganisms. This is because anaerobic conditions are known to stimulate heterotrophic denitrifiers^42,43^ but inhibit aerobic ammonia oxidation microorganisms^44^. Consistent with the previous reports^42,43^, we found a typical heterotrophic denitrifying bacteria *Pseudomonadales* to be enriched in the CMS (Fig. S1). As discussed previously, excessive contamination of the mangrove sediment might reduce the function of mangrove to absorb atmospheric nitrogen but stimulate the ability to buffer anthropogenic nitrogen inputs by discharging them to atmosphere as inert N_2_ gas.

### Contamination promotes microbial capability in generating greenhouse gases

The microbial capacity to generate greenhouse gases was stimulated in contaminated mangrove sediments. It has previously been reported that contamination in mangrove sediments can significantly change the structure of methanogenic microbial communities^6,45,46^. In this new study, we found that contamination in the mangrove sediment also promoted the emission of methane (Supplementary Table S1), and altered methanogenic strategy of methanogens (Fig. 5). There are two types of methanogenic strategy utilized by methanogens; autotrophic and heterotrophic synthesis. Heterotrophic methanogens can metabolize simple substrates (e.g., methanol and acetyl-CoA) into methane via methyltransferase (*mtaA*) and acetyl-CoA decarbonylase/synthase (*cdhc*), whereas autotrophic methanogens can process the reduction of CO_2_ to methane via formylmethanofuran dehydrogenase (*fwdA*)^47,48^. Within the PMS, the consumption of atmospheric methane (Supplementary Table S1) and the abundance of the *fwdA* gene (Fig. 5), indicated that the sediment was relatively oligotrophic and that both methanogens and methanotrophs were functioning to reduce the amount of greenhouse gas in the total budget. In contrast, in the CMS, the abundant *mtaA* gene (Fig. 5), together with the emission of methane gas (Supplementary Table S1), indicated that the excessive amount of contamination not only promoted methane emission but also provided an adequate substrate for heterotrophic methanogens.

As sulfate reduction accounts for almost 100% of the total emission of CO_2_ from mangrove sediments^49^, the enriched levels of sulfate reduction bacteria (Fig. 2) and the sulfate reduction gene (Fig. 4) suggest an increase of CO_2_ emission in the CMS. The enrichment of sulfate reduction related genes in contaminated mangroves were also found in previous metagenomic study, which reported a higher gene abundance of sulfate reduction in Brazilian contaminated mangrove sediments^22^. The lack of difference in the distribution of carbon fixation genes between the various samples might be due to the consistent anaerobic conditions in the mangrove sediment. For example, genes encoding the reductive tricarboxylic acid cycle (Fig. 4), which is an anaerobic carbon fixation metabolism, are the most frequently encoded across samples^50–53^. Therefore, with the increase in CO_2_ generation and the similar carbon fixation capability of microorganisms, the carbon balance in the CMS was bias to releasing instead of fixing. Overall, PMS residing microbes exhibited a strong metabolic ability in absorption of aerial greenhouse gas, while contaminate in mangrove sediments might stimulate bioremediation with an increase of greenhouse gas emission.

### Resistance to anthropogenic pollution

In addition to the input of nitrogen, heavy metals are another major source of pollution in our study areas. Consistent with the previous global investigation^24^, we found that genes associated with the Zn-Pb efflux system and with Co-Zn-Cd resistance were ubiquitous across our terrestrial samples with higher abundant in the contaminated regions; this is in line with the markedly high concentrations of Zn and Pb detected (Fig. 4). Metal contamination is a common phenomenon in polluted mangrove sediments^54,55^. However, the heavy metal resistance genes we detected (Fig. 4) suggest that microbes have developed the capability to minimize the threat of metal pollution.

In our study, organophosphate solubilization-related genes were abundant in the pristine regions. As soluble phosphate is an essential element for the healthy and rapid growth of plants, mangrove plants benefit from sediments that have strong organophosphate solubilization ability^56–58^. The differences we observed in the occurrence of metal resistance and organophosphate solubilizing genes in the contaminated and pristine mangrove sediments, respectively, are likely to be a result of microbial adaptation in battle with heavy metal toxicity in the CMS, and phosphorus limitation in the PMS.

## Conclusions

Through a combination of metagenomic profiling and measurements of sediment quality parameters and methane flux, we have constructed a more comprehensive network analysis for the microbial metabolism in mangrove sediments. Microorganisms exhibit different metabolic capability in pristine and contaminated mangrove sediments as an effect of bioremediation. We found that microorganisms residing in the PMS were more proficient at depositing nitrogen, carbon dioxide, and methane, as well as stimulating organophosphate solubilization; together these could benefit the growth of mangrove plants and reduce the emission of greenhouse gases. In contrast, the microorganisms in the CMS were more proficient in denitrification, greenhouse gas generation, and metal resistance. Overall, contamination in mangrove sediments might have a negative effect on the nutrient deposition ability of microbes, and at the same time it might stimulate bioremediation with an increase of greenhouse gas emission. Determining whether contamination in mangrove sediments might also affect gene expression requires further physiological and transcriptomics investigation.

## Materials and Methods

### Sample collection, genomic dna extraction and sequencing

**Figure 6:**
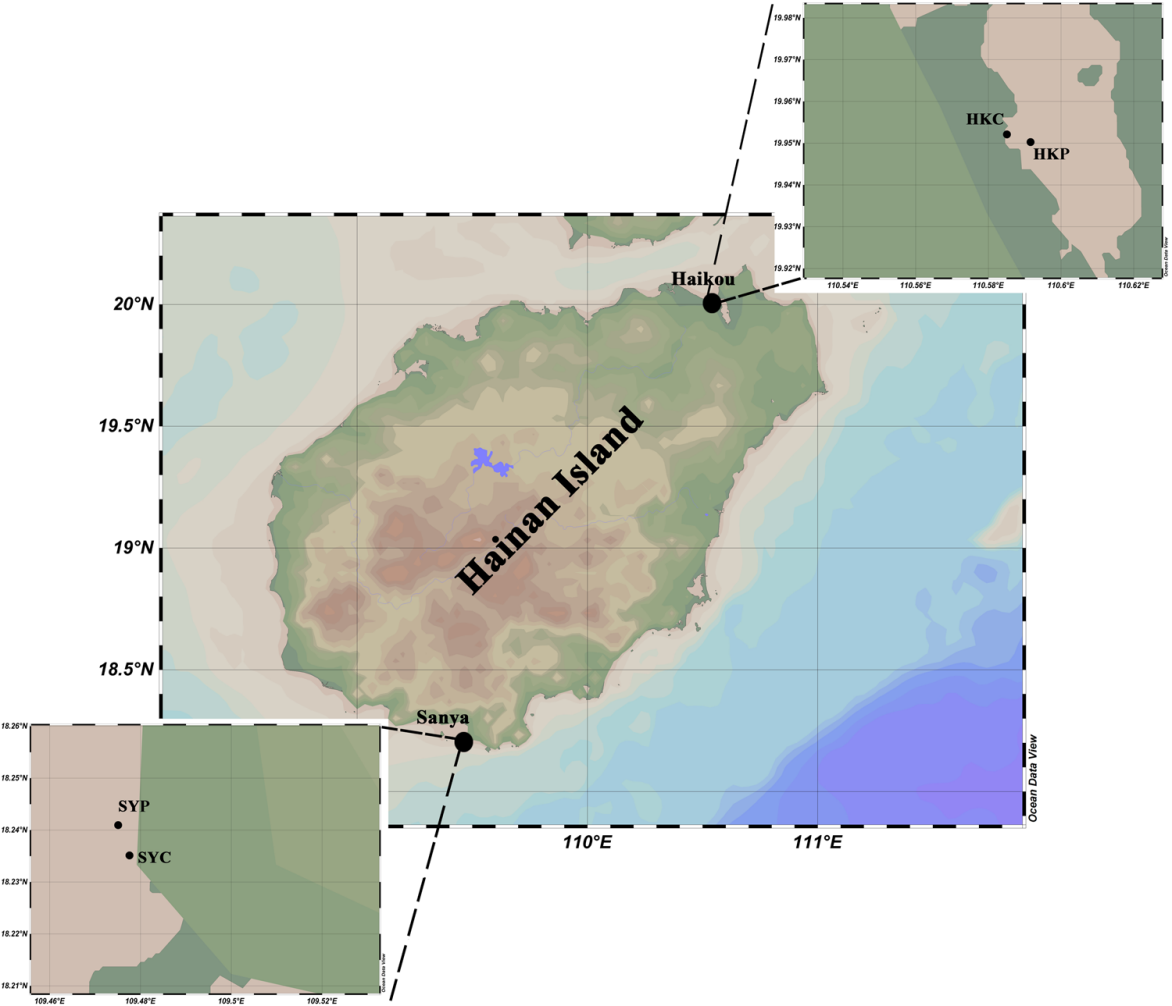
The sampling sites at Hainan Island.

The triplicate mud samples were collected from mangroves located in Haikou (19°194′N, 110°35.214′E) and Sanya (18°15.242′N, 109°30.585′E) in Hainan Island, China, in August 2016 (Fig. 6). Contaminated and pristine mangrove sediment from Haikou were collected from offshore and inshore part of the mangrove forest respectively; whereas, contaminated and pristine sediment from Sanya were sampled from inshore and offshore part of the mangrove forest respectively. At each location, approximately 50 g surface sediment (i.e., from a depth of ~0 cm to 3 cm) was collected and immediately transferred to a 15 ml Falcon tube. The samples were kept on ice while in the field, and then (when back in the laboratory) stored at −80 °C prior to further procedures. Contaminated mangrove sediment (CMS) from Haikou and Sanya was called HKC and SYC, respectively, whereas, pristine mangrove sediment (PMS) from Haikou and Sanya was called HKP and SYP, respectively. DNA extractions of triplicates were performed with the Fast DNA® Spin Kit for Soil (Qbio gene INC), after which the parallel extracts were pooled and shotgun sequencing was performed using an Illumina HiSeq2500 PE 150 bp platform and the HiSeq Cluster Kit v4 kit (San Diego, CA, USA).

### Metagenome assembly

Raw reads were treated using the FASTX-Toolkit package^59^. Reads containing 3 or more ambiguous bases (Ns) and an average quality score <20, or a length <140 bp were removed. The resulting clear paired-end reads were assembled using four different assemblers: IDBA_UD^60^; Velvet^61^; MEGAHIT^62^; and SPAdes^63^, each using their default setting. After comparing the assembly results obtained from the different assemblers, those from the SPAdes software were selected for further analyses.

### Prokaryotic taxonomic assignment and analysis

Screening of SSU rRNAs from the assembled contigs was conducted using Metaxa2^64^. Following the analysis pipe-line previously described^65^, the extracted SSU rRNAs were analyzed using QIIME 1.9.1 against the Silva database (version 123). A filtered OTUs table at 0.1% abundance of each sample was generated with QIIME 1.9.1, and the community structure was visualized with the R software using the ‘vegan’ package^66^.

### Functional annotation

The open reading frames (ORFs) of assembled contigs were predicted using the Prodigal software^67^, with a minimum length of 200 bp. The extracted ORFs were then annotated with the Kyoto Encyclopedia of Genes and Genomes (KEGG) database^68^, the SEED database, and the non-redundant protein (NR) database via DIAMOND^69^ using the following parameters, blastp, k = 1, and an e-value = 10^−7^ to select the best annotation result. For each sample, clean short reads were mapped back to ORFs using Bowtie 2.2.9^70^ to reveal the coverage information for individual functional genes. The web-based analysis tool, KAAS (KEGG Automatic Annotation Server), was used to validate the annotation results from a BLAST (Basic Local Alignment Search Tool) search. In addition, KEGGMAPPER (http://www.genome.jp/kegg/mapper.html) was used to reconstruct the metabolic pathways.

### Chemical analysis of mangrove sediment

For the measurement of nutrients and metals, mangrove sediment samples were collected in acrylic tubes and analyzed in the laboratory on land. To measure the total nitrogen (TN), total phosphate (TP) and total organic carbon (TOC), 1 g dried chips was taken out from the 150 μm mesh filtered mangrove sediment, and 2 gram of catalyst (1 (cupric sulfate, purity >99%): 10 (sodium sulfate, purity >99%)) was then added into the powder grinded from the chips. Then, 2 ml distilled water and 5 ml concentrated sulfuric acid were added to the powder before microwave digestion of the mixture (30 seconds). After digestion, the sample was filtered through a 0.45 μm elutriation membrane, after which the filtrate was analyzed with a FUTURA-II continuously flowing analyzer (Alliance Instruments, France). To determine the salinity, NO_3_^−^, NH_4_^+^, and NO_2_^−^, 30 ml KCl (2 mol/L) was added to the wet sediment, after which the liquid was vibrated for 1 h and then allowed to settle for 30 min before being filtered through a 0.2 μm elutriation membrane. This filtrate was then also analyzed with the FUTURA-II analyzer^71^.

The pH was measured by mixing 5 g sediment with 5 ml water, as reported previously^72^. The sediment was then oven dried for 2 days at 100 °C, after which it was ground up, homogenized and sieved through a 2 mm mesh. Then 7 mL HNO_3_ (72% v/v) and 1 mL H_2_O_2_ (30% v/v) (Fisher Scientific) were added to 0.5 g of each dried sediment sample, and the samples were heated using a closed microwave digestion system (Start D, Milestone) for 90 min until the solutions were clear. The solutions were filtered and then analyzed in triplicate for total concentrations of Zn, Fe, Mn, Cd, and Pb using a 7500 series Inductively Coupled Plasma Mass Spectrometry (ICP-MS) system (Agilent Technologies)^73^. All the measurements for major elements were carried out in triplicates and the standard error was <1%.

### Measurement of sediment methane flux

The flux of methane across the mangrove sediment-atmosphere interface was measured using the Closed Chamber method; the operation and construction of the Perspex chamber (length 50 cm, volume 10 L, enclosing 1000 cm^2^) was as previously reported^74^. In brief, at each location, a Perspex chamber was inserted 5 cm into the sediment for gas collection. The gas in the chamber was extracted with a 10 ml gas-tight syringe every 30 min over a period of 120 min (i.e., at 0, 30, 60, 90, 120 min). The sampled gas was then transferred to 12 ml pre-evacuated exetainers (Labco Limited, USA), and preserved in a small, cold and dry refrigerator (Amoi, china). Within 24 h, the samples were analyzed with gas chromatography (Auto-system, Perkin-Elmer (PE), USA; 3800, Varian, Netherlands), and the methane flux of sampled mangrove sediment was then calculated based on mathematic equations^75^. Measurements were conducted in duplicate for each location, and the final value was averaged.

## Supplementary information

Supplementary material

## Data Availability

The sequencing data supporting the results of this article are available in NCBI Genbank under the accession numbers: PRJNA505165. The predicted open reading frame (ORF) of the assembled contigs were also uploaded to the MG-RAST and annotated with it. The public MG-RAST ID for samples are: mgm4837260.3; mgm4837250.3; mgm4837252.3; mgm4837251.3.
