# Supplementary material for: Comparative metagenomics study reveals pollution induced changes of microbial genes in mangrove sediments
Source: Sci Rep. 2019 Apr 5;9:5739. doi: 10.1038/s41598-019-42260-4 (PMC6450915; doi:10.1038/s41598-019-42260-4)
Supplement: Supplementary file 1 — Supplementary material [file 41598_2019_42260_MOESM1_ESM.doc]

**Comparative metagenomics study reveals pollution induced changes of microbial genes in mangrove sediments**

**Yingdong Li^1^, Liping Zheng^2^, Yue Zhang^2^, Hongbin Liu^1*^, Hongmei Jing^2*^**

*^1^Division of Life Science, The Hong Kong University of Science and Technology, Kowloon, China;*

*^2^CAS Key Laboratory for Experimental Study under Deep-sea Extreme Conditions, Institute of Deep-sea Science and Engineering, Chinese Academy of Sciences, Sanya, China*

**Supplementary materials**

**Table S1** Summary of the environmental conditions and nutrient levels in the four sampling sites

| **Sample** | **Coordinates** | **Temperature**  **(°C)** | **pH** | **Salinity** | **NO_3_^-^** | **NH_4_^+^** | **NO_2_^-^** | **TN** | **TOC** | **TP** | **Zn** | **Fe** | **Mn** | **Cd** | **Ni** | **Pb** | **Methane flux** |
| --- | --- | --- | --- | --- | --- | --- | --- | --- | --- | --- | --- | --- | --- | --- | --- | --- | --- |
|  |  |  |  |  | **(µg/g)** | | | **(mg/g)** | | | **(mg/kg)** | | | | | | **(mg·m^-2^h^-1^)** |
| HKC | 19°57.194＇ | 26.0 | 7.75 | 25.35 | 1.22 | 4.71 | 1.02 | 0.33 | 36.3 | 0.43 | 3577 | 30.3 | 228.4 | 0.37 | 22.7 | 18.6 | 0.58 |
|  | 110°35.214＇ |  |  |  |  |  |  |  |  |  |  |  |  |  |  |  |  |
| HKP | 19°57.167＇ | 25.4 | 7.25 | 27.90 | 0.92 | 7.88 | 0.93 | 0.22 | 32.1 | 0.61 | 2688 | 31.1 | 221.9 | 0.27 | 23.5 | 16.1 | -0.14 |
|  | 110°35.370＇ |  |  |  |  |  |  |  |  |  |  |  |  |  |  |  |  |
| SYC | 18°15.242＇ | 28.5 | 8.20 | 17.10 | 1.53 | 20.1 | 0.83 | 0.35 | 23.7 | 0.37 | 371.1 | 8.64 | 111.6 | 0.16 | 7.72 | 22.9 | 0.28 |
|  | 109°30.585＇ |  |  |  |  |  |  |  |  |  |  |  |  |  |  |  |  |
| SYP | 18°14.392＇ | 29.5 | 7.90 | 35.10 | 1.21 | 11.8 | 0.73 | 0.16 | 7.66 | 0.27 | 205.5 | 4.56 | 126.4 | 0.11 | 4.58 | 7.26 | -0.02 |
|  | 109°30.251＇ |  |  |  |  |  |  |  |  |  |  |  |  |  |  |  |  |

*The value shown in the table is averages of triplicate measurement

**Table S2** Summary of sequence assembly and gene prediction

| **Contig information** | **HKC** | **HKP** | **SYC** | **SYP** |
| --- | --- | --- | --- | --- |
| **Total contigs (≥400bp)** | 231,903 | 207,586 | 172,238 | 198,300 |
| **Size ≥ 1.5 kbp** | 15,126 | 19,213 | 16,530 | 9,358 |
| **Average size of contig(bp)** | 548 | 556 | 631 | 561 |
| **Largest contig (kb)** | 21 | 28 | 26 | 53 |
| **GC content (%)** | 53.78 | 51.10 | 52.56 | 50.32 |
| **Average sequence coverage** | 20.80 | 18.40 | 21.90 | 19.30 |
| **Highest sequence coverage** | 529 | 438 | 512 | 670 |
| **Protein-coding genes(>200 bp)** | 2,897,578 | 2,057,220 | 2,114,595 | 2,905,578 |

**Table S3** Summary of quality control and annotation result

|  | HKC | HKP | SYC | SYP |
| --- | --- | --- | --- | --- |
| Before QC | 19,995,501 | 20,174,812 | 18,476,123 | 20,614,524 |
| After QC | 19,379,214 | 20,156,525 | 18,333,743 | 20,601,447 |
| Annotated genes | 1,775,474 | 1,791,576 | 1,331,047 | 1,704,161 |
| Reads assigned with annotation | 99.44% | 99.51% | 99.64% | 99.63% |


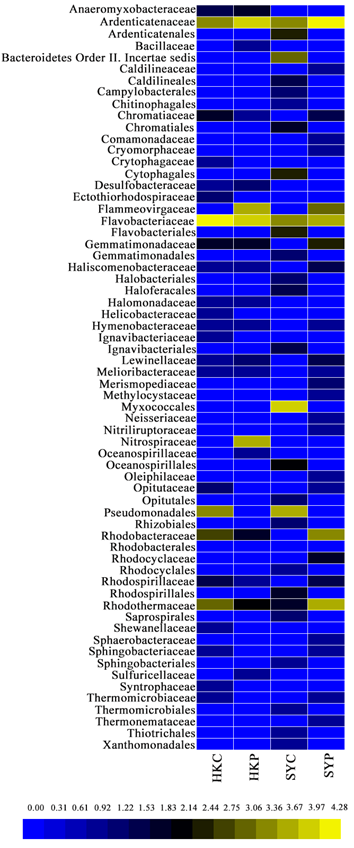


**Figure S1** The *nosZ* gene affiliated microbial taxa at the family level
